# Dyslipidemia Is Positively Associated with Chronic Low Back Pain in Korean Women: Korean National Health and Nutrition Examination Survey 2010–2012

**DOI:** 10.3390/healthcare12010102

**Published:** 2024-01-02

**Authors:** Sunmin Kim, Seon-Mi Lee

**Affiliations:** 1 Department of Anesthesiology and Pain Medicine, Seoul National University Bundang Hospital, Gumi-dong 300, Bundang-gu, Seongnam-si 13620, Republic of Korea; sunmin@snubh.org; 2 Department of Obstetrics and Gynecology, Korea University College of Medicine, 73 Koreadae-ro, Seongbuk-gu, Seoul 02841, Republic of Korea

**Keywords:** dyslipidemia, low back pain, Korean women, hypertension, diabetes, chronic diseases

## Abstract

Background: This study aimed to evaluate the association between chronic low back pain (CLBP) and chronic diseases, such as hypertension, diabetes, and dyslipidemia. Methods: This study was a retrospective analysis using data from the Korea National Health and Nutrition Examination Survey (KNHANES) between 2010–2012 and included women who answered a questionnaire in the KNHANES asking whether they had low back pain for ≥3 months. Participants were divided into non-CLBP and CLBP groups. For statistical analysis, the Student’s *t*-test, chi-square test, Fisher’s exact test, and logistic regression analysis were performed using SPSS. Results: Of 5961 participants, the non-CLBP group comprised 4098 women and the CLBP group comprised 1863. Adjusted logistic regression model revealed that dyslipidemia was positively associated with CLBP (odds ratio, 1.32; 95% confidence interval, 1.140, 1.530; *p* < 0.001). However, hypertension and diabetes were not associated with CLBP. Conclusions: Our results suggest that proper treatment of dyslipidemia may contribute to lowering the risk of CLBP later in life.

## 1. Introduction

According to statistics reported by the World Health Organization in 2020, 619 million individuals suffered from low back pain worldwide, which is expected to increase to approximately 843 million by 2050 [1]. A meta-analysis of the burden of low back pain in developed countries through 2023 found that the average annual direct cost per population ranged between USD 3.4–3.6 billion. Furthermore, in 2015, South Korea suffered an economic loss of approximately USD 6.6 billion due to low back pain. Since 2010, low back pain has ranked as the second most socioeconomically burdensome disease in South Korea among many conditions [2]. The impact of low back pain not only extends into our daily lives; it is also associated with economic losses for the country.

In Korea, the incidence of low back pain in men and women is 11.8% and 24.5%, respectively, so the incidence of low back pain in women tends to be much higher than in men [3]. Women have a higher sensitivity to pain than men, and hormonal changes due to menstrual cycle fluctuations contribute to this increased pain sensitivity. In addition, biological changes caused by increased abdominal weight gain in perimenopausal women can also contribute to low back pain, making women more vulnerable to the occurrence of LBP compared to men [4]. For this reason, this study focused on Korean women as they are at particularly high risk for low back pain in the overall Korean population.

Low back pain is categorized as acute if it lasts <6 weeks, subacute if it lasts between 4 and 12 weeks, or chronic if it lasts for >12 weeks [5]. The most common cause of low back pain is muscle strain or ligament sprains. Low back pain caused by the aforementioned causes is usually classified as acute or subacute and improves within a few weeks if the patient does not overexert themselves and rests [6]. However, despite sufficient stability, self-care of chronic low back pain (CLBP) can be difficult. CLBP is not only caused by injuries to muscles and ligaments; it is often accompanied by other causes, such as a herniated disc, compression fracture, or spondylolisthesis [6]. The aforementioned conditions can be caused by bone health complications, which can be affected by the patient’s lifestyle and underlying diseases.

Among the underlying diseases that can affect CLBP, we focused on three major chronic diseases: hypertension, diabetes, and dyslipidemia. Hypertension can also cause aortic aneurysms. In particular, because the abdominal aorta is adjacent to the lower spinal area, an abdominal aortic aneurysm can cause CLBP due to nerve compression in the spinal cord [7]. In diabetes, when blood glucose levels are elevated, insulin and stress hormones are also elevated, creating an inflammatory environment. When this inflammatory condition develops around the lower spinal discs, it induces CLBP [8,9]. Abnormal blood lipid levels, such as high triglyceride (TG) and low-density lipoprotein (LDL) cholesterol, can induce atherosclerosis in the lumbar vessels, resulting in CLBP [10]. Based on the aforementioned mechanisms, we hypothesized that there is a positive relationship between CLBP and hypertension, diabetes, and dyslipidemia. Several studies have evaluated the association between hypertension, diabetes, dyslipidemia, and CLBP. Studies have reported a significant positive relationship between hypertension and CLBP [11,12,13], whereas others have indicated a negative relationship [14,15,16]. Some studies have found a meaningful association between diabetes and CLBP [9,17,18,19], whereas others have reported no relationship [20,21]. Similarly, some studies have suggested a positive relationship between dyslipidemia and CLBP [10,22,23,24,25], whereas others have found no association between them [23,26,27]. Based on the results of these published studies, the association between CLBP and chronic diseases is still inconsistent. Additionally, most existing studies have been conducted in Western populations, and only a few studies have been conducted among Korean women. Therefore, this study aimed to evaluate the association between LBP and chronic diseases in Korean women.

## 2. Materials and Methods

### 2.1. Study Participants and Design

This cross-sectional study used KNHANES data gathered between 2010 and 2012. The KNHANES has been conducted every three years since 1998 to understand the health level, health behavior, food, and nutrition of the Korean population. All steps included in the questionnaire, including height, weight, waist circumference, body mass index (BMI), and the health questionnaire, were conducted by well-trained medical staff.

**Figure 1:**
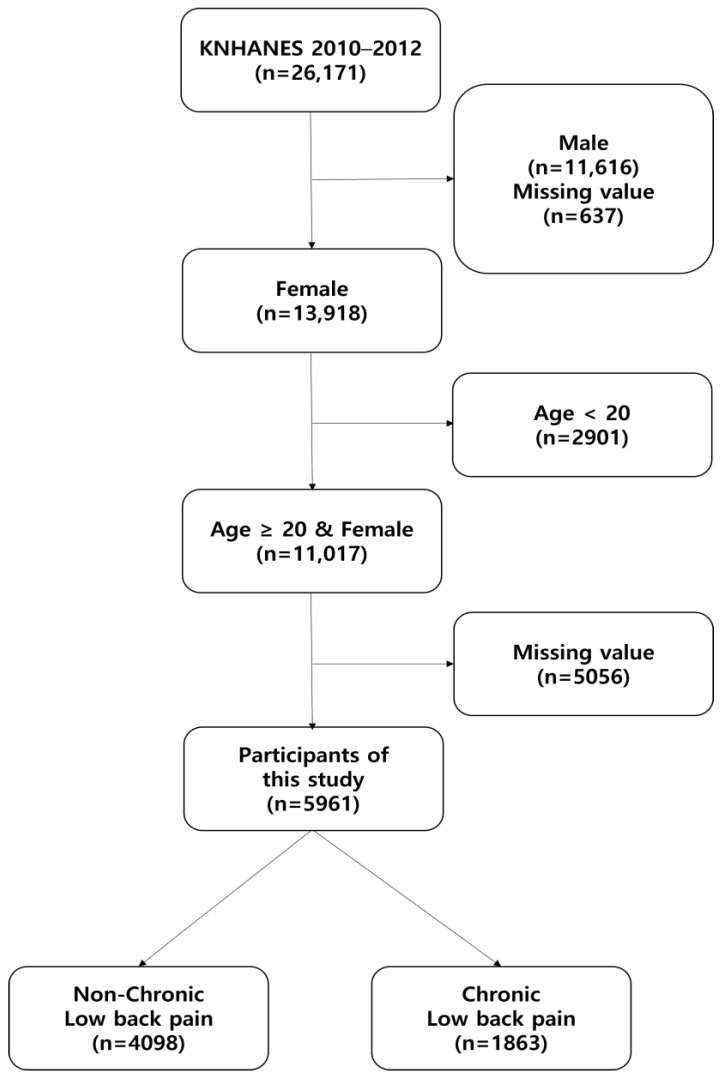
Diagram of participants included in the final analysis. Note: KNNHANES, Korean National Health and Nutrition Examination Survey.

Inclusion criteria for this study was women aged ≥ 20 years who responded to a questionnaire asking if they had CLBP for >3 months. Exclusion criteria were men, women < 20 years of age, and missing values (e.g., unfilled or missing data). Of the missing values excluded from the study, 4058 were women who were pregnant, and none were lactating after childbirth. The final participants did not include any women who were pregnant or breastfeeding after childbirth. As a result, according to our criteria, 5961 participants were selected from the 26,171 individuals surveyed between 2010 and 2012 (Figure 1). The participants were divided into two groups: non-CLBP and CLBP.

### 2.2. Study Variables

The KNHANES includes the results of health surveys, asking for information on medical use, health examination results, education and economic activities, obesity, weight control, drinking, smoking, mental health, physical activity, and women’s health; examination surveys, including blood pressure measurements, blood tests, and body measurements; and nutrition surveys, including investigating dietary habits and nutritional information.

Body mass index was obtained through the following formula using the measured height and weight (kg/m^2^) and classified as underweight (BMI < 18.5 kg/m^2^), normal weight (18.5 kg/m^2^ ≤ BMI < 23 kg/m^2^), pre-obesity (23 kg/m^2^ ≤ BMI < 25 kg/m^2^), and obesity (BMI ≥ 25 kg/m^2^). Waist circumference was measured at the narrowed part between the rib cage and iliac crest after normal expiration. This study categorized personal income levels into four quartiles based on the average monthly household income ([monthly overall household income] [household size]^−0.5^): Q1. Low, Q2. Low-intermediate, Q3. Upper intermediate, and Q4. High. The degree of education was classified as elementary school, middle school, high school, and college or higher. Drinking was divided into five categories based on the frequency of drinking: no drinking at all, less than once a month, once a month, once a week, and almost every day. Smokers were classified as non-smokers or smokers based on the contents of a questionnaire that surveyed adults for smoking throughout their lives. In the questionnaire asking whether or not they took oral contraceptives (OC), those who answered “yes” were included in a yes group of OC use, and those who answered “no” were included in a no group. A questionnaire assessed the number of days of walking exercise per week to understand physical activity, which was classified as follows: (1) not at all; (2) 1 d; (3) 2 d; (4) 3 d; (5) 4 d; (6) 5 d; (7) 6 d; and (8) 7 d (every day). We compared the information on the frequency of walking exercises by classifying the participants into a group that walked less than one day a week, a group that walked 1–3 days a week, and a group that walked 4–7 d a week. In addition to walking, the survey includes a questionnaire asking how many strength training exercises the participant completes in a week, which is categorized as follows: (1) not at all; (2) 1 d; (3) 2 d; (4) 3 d; (5) 4 d; (6) 5 d; (7) 6 d; and (8) 7 d (every day). Based on the aforementioned items, this study categorized strength training as less than 1 d per week, 1–3 days per week, and ≥4 d per week. The dietary habits of the participants were identified using survey items that assessed the quantity and quality of food intake; (1) eating a sufficient amount and variety of food; (2) eating a sufficient amount of food but not eating various foods; (3) sometimes not having enough food to eat due to economic difficulties; and (4) often not having enough food because of economic difficulties.

Blood samples were collected from the antecubital vein in the morning after overnight fasting. Total cholesterol, TG, and plasma glucose levels were measured using a Labospect008AS (Hitachi, Tokyo, Japan). Additionally, HbA1c levels were measured using a Tosoh G8 (Tosoh, Tokyo, Japan).

The participants were divided into two groups according to their hypertension status: normal and hypertensive. Normal was defined as systolic blood pressure (SBP) < 120 mmHg and diastolic blood pressure (DBP) < 80 mmHg, answering “no” to the question “Have you been diagnosed with hypertension by doctor?”, or answering “no” to the question “Do you currently suffer from hypertension?” Hypertension was defined as SBP ≥ 140 mmHg or DBP ≥ 90 mmHg, answering “yes” to the question “Have you been diagnosed with hypertension by doctor?”, or answering “yes” to the question “Do you currently suffer from hypertension?” The participants were classified into two groups according to their diabetes status: normal and diabetic. Normal was defined as fasting glucose level < 100 mg/dL or HbA1c level < 5.7%, answering “no” to the question “Have you been diagnosed with diabetes by doctor?”, or answering “no” to the question “Do you currently suffer from diabetes?” Diabetes was defined as fasting glucose level ≥ 126 mg/dL or HbA1c ≥ 6.5%, answering “yes” to the question “Have you been diagnosed with diabetes by doctor?”, or answering “yes” to the question “Do you currently suffer from diabetes?” If the cholesterol level was <240 mg/dL and TG level was <200 mg/dL, if they answered “no” to the question “Have you been diagnosed with dyslipidemia by doctor?”, or if they answered “no” to the question “Do you currently suffer from dyslipidemia?”, they were defined as a normal dyslipidemia status group. If the cholesterol level was ≥240 mg/dL or TG level ≥ 200 mg/dL, if they answered “yes” to the question “Have you been diagnosed with dyslipidemia by doctor?”, or if they answered “yes” to the question “Do you currently suffer from dyslipidemia?”, they were defined as a dyslipidemia group.

### 2.3. Statistical Analysis

The KNHANES data were extracted using a two-stage stratified cluster sampling design rather than a simple random sampling design method; therefore, statistical analysis was conducted based on the complex sample analysis method. An integrated weight was applied to integrate the three years of data, and one data point was produced. Comparisons of continuous variables between the two groups, according to LBP status, were performed using the Student’s *t*-test, and categorical variables were analyzed using the chi-square test or Fisher’s exact test. To analyze the risk of LBP and non-LBP, a univariate analysis was performed for each investigated variable. Multivariate logistic regression analysis was performed to evaluate the risk of LBP according to chronic disease status, such as hypertension, diabetes, and dyslipidemia. We considered 10 confounding variables (age, weight, height, waist circumference, individual income, education level, history of taking OC, number of days of walking exercises per week, number of days of strength exercises per week, and dietary circumstances), which were adjusted in the multivariate logistic regression analysis. All analyses were performed using SPSS Statistics for Windows version 25.0 (SPSS Inc., Chicago, IL, USA). For all the analyses, *p*-values < 0.05 were considered statistically significant.

### 2.4. Ethics

We conducted a retrospective study using the KNHANES data collected after receiving Institutional Review Board (IRB) approval from the Korea Center for Disease Control and Prevention. Therefore, approval was not required from the IRB.

## 3. Results

The mean age of the study population was 61.81 ± 10.53 years; 59.78 ± 10.17 years for non-CLBP participants, and 66.26 ± 9.91 years for CLBP participants. Of the 5961 participants, 4098 were in the non-CLBP group, and 1863 were in the CLBP group. A comparison of the two groups’ characteristics is presented in Table 1. Statistically significant differences were observed between the non-CLBP and CLBP groups in terms of age, body measurements (weight, height, BMI, and waist circumference), individual income, education level, alcohol consumption, OC use, degree of walking exercise, degree of strength exercise, dietary circumference, hypertension status, diabetes status, and dyslipidemia status. In the CLBP group, BMI (especially overweight, obese levels of BMI) (*p* = 0.016), high waist circumference (*p* < 0.001), low individual income (*p* < 0.001), elementary school or lower (*p* < 0.001), alcohol consumption less than once a month (*p* < 0.001), OC use (*p* < 0.001), and the proportion of individuals who did not perform walking and strength exercises at least one day a week (*p* < 0.001) were higher than those in the non-CLBP group. The present rates of all chronic diseases, including hypertension (*p* < 0.001), diabetes (*p* < 0.001), and dyslipidemia (*p* < 0.001), were significantly different between the non-CLBP and CLBP groups.

The univariate analysis results regarding the risk of CLBP compared to non-CLBP participants are shown in Table 2. Significant factors associated with the risk of CLBP included older age (odds ratio [OR], 1.07; 95% confidence interval [CI], 1.059, 1.071; *p* < 0.001), low body weight (OR, 0.99; 95% CI, 0.984, 0.997; *p* = 0.003), short height (OR, 0.94; 95% CI, 0.932, 0.949; *p* < 0.001), high waist circumference (OR, 1.03; 95% CI, 1.020, 1.032; *p* < 0.001), lower individual income (OR, 0.76; 95% CI, 0.655, 0.886; *p* < 0.001 in Q2; OR, 0.61; 95% CI, 0.521, 0.709; *p* < 0.001 in Q3; and OR, 0.52; 95% CI, 0.442, 0.606; *p* < 0.001 in Q4), low education level (OR, 0.47; 95% CI, 0.401, 0.558; *p* < 0.001 in middle school; OR, 0.28; 95% CI, 0.234, 0.323; *p* < 0.001 in high school; and OR, 0.18; 95% CI, 0.140, 0.241; *p* < 0.001 in college or higher), OC use (OR, 1.36; 95% CI, 1.188, 1.546; *p* < 0.001), less walking (OR, 0.51; 95% CI, 0.439, 0.596; *p* < 0.001 in 1–3 days/week; OR, 0.62; 95% CI, 0.539, 0.705; *p* < 0.001 in ≥4 days/week) and strength exercise for a week (OR, 0.48; 95% CI, 0.388, 0.590; *p* < 0.001 in 1–3 days/week; OR, 0.72; 95% CI, 0.559, 0.928; *p* = 0.011 in ≥4 days /week), inability to consume sufficient and diverse food (OR, 1.60; 95% CI, 1.412, 1.803; *p* < 0.001 in eating enough but not diverse food; OR, 3.33; 95% CI, 2.522, 4.383; *p* < 0.001 in eating poorly sometimes; and OR, 4.48; 95% CI, 2.402, 8.363; *p* < 0.001 in eating poorly frequently), hypertension (OR, 1.86; 95% CI, 1.664, 2.080; *p* < 0.001), diabetes (OR, 1.50; 95% CI, 1.276, 1.758; *p* < 0.001), and dyslipidemia (OR, 1.41; 95% CI, 1.232, 1.608; *p* < 0.001).

We performed multivariate logistic regression analysis by adjusting age, weight, height, waist circumference, OC use, individual income, education level, number of days of walking exercise per week, number of days of strength exercise per week, and dietary circumferences factors, which were statistically significant factors in the univariate logistic regression. Among the aforementioned factors, model 1 adjusted for age, weight, height, waist circumference, and OC use, model 2 adjusted for individual income and education level in addition to the factors adjusted in model 1, and model 3 adjusted for factors such as number of days of walking exercise per week, number of days of strength exercise per week, and dietary circumference in addition to the factors adjusted in model 2. The results for the multivariate logistic regression analysis are presented in Table 3. Multivariate logistic regression analysis showed that the risk of CLBP was significantly higher in patients with dyslipidemia in models 1, 2, and 3 (OR, 1.25; 95% CI, 1.088, 1.439; *p* = 0.002 in model 1; OR, 1.32; 95% CI, 1.144, 1.522; *p* < 0.001 in model 2; OR, 1.32; 95% CI, 1.140, 1.530; *p* < 0.001 in model 3). Hypertension and diabetes were not associated with CLBP risk in models 1, 2, or 3.

According to the results of the multivariate logistic regression analysis shown in Table 3, models 1, 2, and 3 confirm a significant positive relationship between CLBP and dyslipidemia. Since dyslipidemia is more likely to occur in obese individuals, it is necessary to evaluate the association between LBP and dyslipidemia according to participants’ BMI. Therefore, we performed a multivariate logistic regression analysis in which all participants were categorized into BMI underweight (BMI < 18.5), normal (18.5 ≤ BMI < 23), overweight (23 ≤ BMI < 25), and obese groups (BMI ≥ 25), adjusting for age, weight, height, waist circumference, OC use, individual income, education level, number of days of walking exercise per week, number of days of strength exercise per week, and dietary circumstances factors. The results are presented in Table 4. Obesity may predispose individuals to the development of dyslipidemia, and this influence was reflected in the present study, in which there was no association between dyslipidemia and CLBP in the underweight, normal, and overweight BMI groups, but dyslipidemia was associated with a significantly increased risk of CLBP in participants with obese BMI.

Multivariate logistic regression analysis confirms a significant positive relationship between CLBP and dyslipidemia. To examine whether these results apply when categorized by age, we performed a multivariate logistic regression analysis by dividing all participants into two groups: those under 60 and those over 60, based on the average age of 60. The results are presented in Table 5. The presence of dyslipidemia among chronic diseases was associated with a significantly increased risk for CLBP in both the under-60 and over-60 age groups.

## 4. Discussion

In this retrospective study, we found that women with dyslipidemia had a significantly higher risk of developing CLBP. In particular, the risk of CLBP was significantly increased in the presence of dyslipidemia in obese participants among all participants, and there was a positive relationship between dyslipidemia and CLBP in all participants categorized by age who were in their 60s. However, contrary to our assumption, hypertension and diabetes were not associated with the risk of CLBP. To the best of our knowledge, this study is one of the few to investigate the relationship between CLBP and chronic diseases among Korean women.

Based on the mechanism that CLBP can be caused by hypertension-induced aneurysms in the aorta adjacent to the lumbar region [7]. One study reported a positive relationship between hypertension and CLBP [11], another found a significant association between hypertension and severe CLBP [23], and an additional study reported a significantly increased risk of CLBP in a group of men with hypertension [13]. Based on the above, we assumed that there would be a positive relationship between hypertension and CLBP. However, we did not find this result in our study. Among the previously published studies, a prospective study by Hemingway et al. found no association between hypertension and back pain (OR, 1.07; 95% CI, 0.7, 1.6 in men; OR, 1.49; 95% CI, 0.8, 2.6 in women), which is consistent with our findings [28]. Contrary to our hypothesis, other studies have reported a negative relationship between hypertension and LBP. In a cross-sectional study by Bae et al., the risk of CLBP in South Koreans individuals was reduced in those with hypertension with SBP ≥ 140 mmHg (OR, 0.81; 95% CI, 0.70, 0.94) and DBP ≥ 90 mmHg (OR, 0.73, 95% CI, 0.63, 0.85) [14]. Heuch’s cross-sectional study of the Norwegian population showed that a 100 mmHg increase in SBP was associated with significantly reduced risk of CLBP in women (OR, 0.95; 95% CI, 0.92, 0.99; *p* = 0.005), whereas no association was observed between SBP and CLBP in men [15]. Similarly, a study by Hagen in Norway reported a decreased incidence of CLBP in a hypertension setting with SBP ≥ 140 mmHg (OR, 0.7; 95% CI, 0.6, 0.8) and DBP ≥ 90 mmHg (OR, 0.7, 95% CI, 0.6, 0.8) [16]. Studies reporting a negative relationship between hypertension and CLBP support the following mechanisms: in an environment of hypertension, plasma endorphins, which are endogenous opioids, are increased, and as a result, the threshold for pain is increased; thus, the perception of pain itself tends to decrease, leading to a decrease in CLBP [14]. However, a clear mechanism explaining the relationship between hypertension and CLBP has not yet been established. In some cases, such as in our study, no association between hypertension and CLBP was observed. Therefore, further studies are needed to clarify this.

No significant relationship was found between diabetes mellitus (DM) and LBP in this study. Similarly, a cross-sectional and longitudinal study by Dario et al. reported no significant association between type 2 DM and CLBP (OR, 1.10; 95% CI, 0.54, 2.22) in a logistic regression analysis after adjusting for genetic and external environmental factors, and there was no association between type 2 DM and future CLBP in a longitudinal analysis [20]. A cohort study of twins in the United Kingdom found no significant association between type 2 DM and CLBP [21]. However, other studies have reported a positive association between DM and LBP [17,18,19]. A retrospective study of 139,002 individuals in Germany found an increased risk of chronic CLBP in patients with type 2 DM (HR, 1.23; 95% CI, 1.13, 1.36) [17], and a retrospective single center study reported a significantly higher CLBP score in patients with type 2 DM for > 10 years (CLBP score of type 2 DM ≥ 10 years, 3.83 ± 0.86; CLBP score of type 2 DM < 10 years, 3.49 ± 0.74; *p* < 0.05) [19]. A meta-analysis conducted by Pozzobon et al. also found a significantly higher risk of CLBP in patients with DM (OR, 1.35; 95% CI, 1.20, 1.52; *p* < 0.001) [18]. They argued that DM can lead to an increased incidence of LBP based on the following mechanisms: Type 1 DM, which is caused by reduced insulin secretion as a result of damage to pancreatic beta cells, creates an environment of chronic hyperglycemia and IL-1 beta stress. Type 2 DM, which is caused by insulin resistance, leads to hyperglycemia, hyperinsulinemia, and increased blood fatty acid levels. Types 1 and 2 DM increase glucose uptake by increasing the expression of glucose transporters to control high blood glucose levels. This results in oxidative stress, which can damage perivascular tissues [8,9]. Additionally, this hyperglycemic environment can cause calcifying lesions in blood vessels, thereby reducing blood flow. In the intervertebral disc area, reduced blood flow not only reduces the supply of oxygen and nutrients but also inhibits the removal of waste products, such as lactic acid, which can contribute to LBP development [8,9]. However, DM does not exist in isolation, but can coexist with obesity, as well as sarcopenia, a condition characterized by decreased muscle mass and strength. In a cross-sectional study using data from the National Health Interview Survey in the United States between 1997 and 2004, obesity with a BMI of >35 kg/m^2^ was associated with a nine-fold increase in the incidence of DM compared to underweight individuals with a BMI of <18 kg/m^2^ [29]. A prospective cohort study evaluating the association between BMI and CLBP reported a significantly increased risk of CLBP with a BMI of >30 kg/m^2^ compared to a BMI of <25 kg/m^2^ (OR, 1.34; 95% CI, 1.08, 1.67 in men; OR, 1.22; 95% CI, 1.03, 1.46 in women) [30]. Additionally, several studies have reported a significant increase in the incidence of CLBP in patients with sarcopenia compared with that of patients without sarcopenia [31,32,33]. In light of these findings, it is difficult to conclude that DM is directly associated with CLBP, as obesity and sarcopenia, which can coexist with DM, may be significant risk factors for an increased incidence of CLBP.

**Figure 2:**
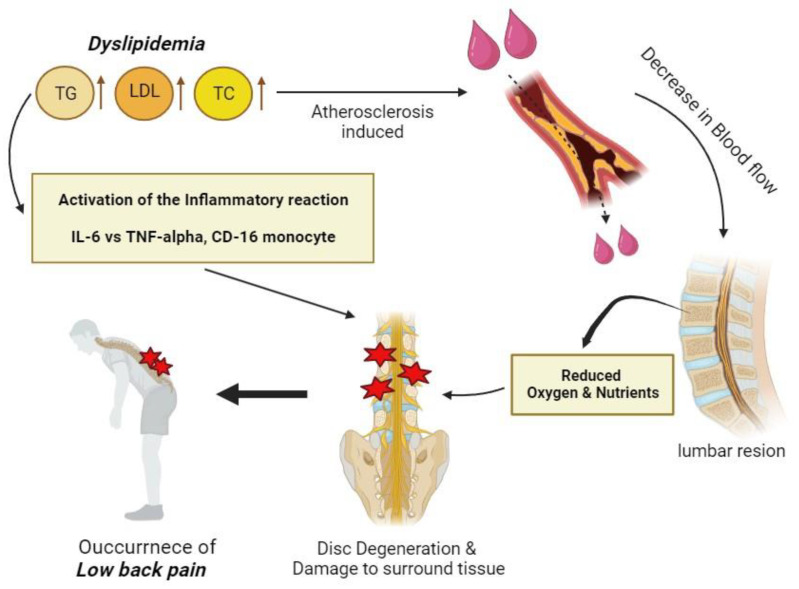
Schematic representation of the mechanism between dyslipidemia and low back pain. Note: TG, triglyceride; LDL, low-density lipoprotein; TC, total cholesterol; IL, interleukin; TNF, tumor necrosis factor.

The association between CLBP and dyslipidemia has been identified in previous studies. Yoshimoto et al. conducted a cross-sectional study of middle-aged Japanese individuals and performed a multivariate logistic regression analysis adjusting for age, BMI, and lifestyle factors. The results showed that high-density lipoprotein (HDL) < 40 mg/dL (OR, 1.34; 95% CI, 1.20, 1.48 in men; OR, 1.32; 95% CI, 1.02, 1.72 in women) and LDL/HDL ratio ≥ 2.5 (OR, 1.17; 95% CI, 1.09, 1.26 in men; OR, 1.15; 95% CI, 1.03, 1.29 in women) significantly increased the risk of CLBP in both men and women [10]. Yuan et al. found that Chinese individuals with high TG ≥ 6.2 mg/dL (OR, 1.775; 95% CI, 1.209, 2.606) and high LDL ≥ 4.1 mmol/L (OR, 1.818; 95% CI, 1.123, 2.943) tended to have a higher risk of developing lumbar intervertebral disc degeneration, a known cause of LBP [22]. Similarly, a retrospective study of 320 Chinese patients and a case-control study of 105 Koreans by Haung et al. found that higher total cholesterol and TG levels were associated with increased lumbar intervertebral disc degeneration and a significantly higher incidence of LBP [23,24]. A case-control study of 269 patients found an increased risk of intervertebral disc degeneration in the setting of dyslipidemia with high TG (OR, 1.753; 95% CI, 1.151, 2.699; *p* = 0.009), high LDL (OR, 1.952; 95% CI, 1.530, 2.490; *p* < 0.001), or high total cholesterol (OR, 3.580; 95% CI, 2.182, 55.876; *p* < 0.001). Disc degeneration was associated with a significantly higher incidence of lumbar disc herniation, and consequently CLBP [25]. Our study also confirmed the association between LBP and dyslipidemia. Although the mechanisms linking low back pain and dyslipidemia are not fully understood, several hypotheses suggest a connection, as shown in Figure 2. Dyslipidemia increases the levels of TG and LDL in blood, resulting in an overall high total cholesterol level. This leads to atherosclerosis of blood vessels, which reduces blood flow. If blood vessels in the lumbar region are exposed to this environment, the oxygen and nutrient supply to the musculoskeletal tissue surrounding the lumbar area is reduced. Insufficient oxygen and nutrient supplies can cause damage and degeneration of various tissues, including soft tissues, leading to disc degeneration in the lumbar region [10]. Additionally, in the environment of dyslipidemia, the secretion of pro-inflammatory cytokines, such as IL-6 is activated, and to counteract this, the secretion of CD-16 monocytes and TNF-alpha is also increased, creating an environment in which the inflammatory response is activated [34,35]. This inflammatory environment can accelerate disc degeneration; consequently, dyslipidemia contributes to an increased incidence of low back pain.

This study has some limitations. First, this was a cross-sectional study, and we were unable to perform a longitudinal follow-up. In addition, since this study was designed as a cross-sectional study, it is possible to analyze the association by obtaining both the cause and result at one point in time, but there is a limitation that it is difficult to clearly establish a causal relationship through this study. Second, participants were excluded from the study if they did not complete the questionnaire or if they were systemically missing values. In this study, 5056 participants were excluded due to missing values, which means that more than 45% of women aged 20 and older (n = 11,017) were excluded. As a result, there may be exclusion bias due to the large number of excluded participants. Third, because the survey responses depended on each individual’s memory, a potential recall bias cannot be excluded. Fourth, this study used the KNHANES data, which consists of a questionnaire, so it is possible to determine the presence of CLBP, but it is not possible to obtain information on the severity and duration of CLBP or medication. Therefore, this study cannot evaluate the association between the duration and severity of chronic CLBP and chronic diseases. Nevertheless, the strength of our study was that 3 years of large national representative sample data were used and analyzed to determine the association between CLBP and various factors; additionally, statistical analysis was performed by adjusting for confounding variables. Consequently, we confirmed an association between dyslipidemia and CLBP. In the future, a longitudinal follow-up study is required to evaluate how correcting dyslipidemia contributes to lowering the risk of CLBP. Furthermore, although this study only included Korean women, further research is needed to identify whether the association between dyslipidemia and CLBP also applies to women of other ethnicities, not just Korean women.

## 5. Conclusions

In our study, dyslipidemia was positively associated with CLBP, including hypertension, diabetes, and dyslipidemia, among the three major chronic diseases in Korean women. Therefore, encouraging proper treatment of dyslipidemia in women may contribute to lowering the potential risk of CLBP in the future.

## Figures and Tables

**Table 1:** Characteristics of the study participants according to chronic low back pain status.

	Total (N = 5961)	Non-CLBP (N = 4098)	CLBP (N = 1863)	*p*-Value
Age (years), M ± SD	61.81 ± 10.53	59.78 ± 10.17	66.26 ± 9.91	<0.001
Weight (kg), M ± SD	57.26 ± 8.82	57.29 ± 8.55	56.76 ± 9.35	0.004
Height (cm), M ± SD	153.91 ± 6.07	154.60 ± 5.84	152.38 ± 6.30	<0.001
BMI, N (%)				0.016
BMI < 18.5	147 (2.5%)	98 (2.4%)	49 (2.6%)	
18.5 ≤ BMI < 23	2154 (36.1%)	1509 (36.8%)	645 (34.6%)	
23 ≤ BMI < 25	1519 (25.5%)	1073 (26.2%)	446 (24.1%)	
BMI ≥ 25	2141 (35.6%)	1418 (34.6%)	723 (38.7%)	
Waist circumference (cm), M ± SD	81.58 ± 9.34	80.00 ± 9.11	83.12 ± 9.65	<0.001
Income, N (%)				<0.001
Q1	1474 (24.6%)	908 (22.1%)	566 (31.3%)	
Q2	1503 (25.3%)	1004 (24.5%)	499 (26.4%)	
Q3	1526 (25.5%)	1084 (26.5%)	442 (22.7%)	
Q4	1458 (25.1%)	1102 (26.9%)	356 (19.6%)	
Education, N (%)				<0.001
Less than elementary school	3229 (54.0%)	1880 (45.5%)	1349 (72.6%)	
Middle school	900 (15.1%)	669 (16.4%)	231 (12.4%)	
High school	1293 (21.9%)	1075 (26.5%)	218 (11.6%)	
College or higher	539 (9.0%)	474 (11.6%)	65 (3.4%)	
Alcohol consumption,				<0.001
N (%)				
Not drinking at all	3838 (61.0%)	2312 (59.0%)	1526 (66.4%)	
Less than once a month	1242 (20.3%)	1055 (20.5%)	187 (19.8%)	
About once a month	530 (10.3%)	448 (11.2%)	82 (7.8%)	
About once a week	275 (7.0%)	230 (7.8%)	45 (4.4%)	
Almost every day	76 (1.4%)	53 (1.5%)	23 (1.6%)	
Smoking, N (%)				0.187
Nonsmoker	5502 (92.5%)	3794 (92.8%)	1708 (91.8%)	
Smoker	459 (7.5%)	304 (7.2%)	155 (8.2%)	
Oral contraceptive use,				<0.001
N (%)				
No	4707 (79.1%)	3301 (80.7%)	1406 (75.5%)	
Yes	1254 (20.9%)	797 (19.3%)	457 (24.5%)	
Number of days of walking exercise per week				<0.001
0 day/week	1365 (22.9%)	808 (19.7%)	557 (29.9%)	
1–3 days/week	1706 (28.6%)	1260 (30.8%)	446 (23.9%)	
≥4 days/week	2890 (45.5%)	2030 (49.5%)	860 (46.2%)	
Number of days of strength exercise per week				<0.001
0 day/week	4998 (84.0%)	3347 (81.8%)	1651 (89.0%)	
1–3 days/week	629 (10.4%)	506 (12.3%)	123 (6.4%)	
≥4 days/week	334 (5.6%)	245 (5.9%)	89 (4.6%)	
Dietary circumstances,				<0.001
N (%)				
Eating enough and				
varied food	2348 (39.4%)	1755 (43.3%)	593 (30.9%)	
Eating enough but				
not diverse food	3261 (55.7%)	2142 (53.4%)	1119 (60.8%)	
Eating poorly				
Sometimes	304 (4.1%)	180 (2.9%)	124 (6.8%)	
Eating poorly				
frequently	48 (0.8%)	21 (0.4%)	27 (1.5%)	
Hypertension status,				<0.001
N (%)				
Normal	3663 (61.4%)	2709 (66.1%)	954 (51.2%)	
Hypertension	2298 (38.6%)	1389 (33.9%)	909 (48.8%)	
Diabetes status, N (%)				<0.001
Normal	5235 (87.8%)	3657 (89.2%)	1578 (84.7%)	
Diabetes	726 (12.2%)	441 (10.8%)	285 (15.3%)	
Dyslipidemia status,				<0.001
N (%)				
Normal	4774 (80.1%)	3354 (81.8%)	1420 (76.2%)	
Dyslipidemia	1187 (19.9%)	744 (18.2%)	443 (23.8%)	

Note: Values are presented mean ± standard deviation or N (%); M ± SD, mean ± standard deviation; Q, quarter; CLBP, chronic low back pain; BMI, body mass index.

**Table 2:** Univariate logistic regression analysis results of risks for chronic low back pain compared to those with non-chronic low back pain.

Risk for Chronic Low Back Pain Compared to That of Non-Chronic Low Back Pain			
	OR	95% CI	*p*-Value
Age (years)	1.07	(1.059, 1.071)	<0.001
Weight (kg)	0.99	(0.984, 0.997)	0.003
Height (cm)	0.94	(0.932, 0.949)	<0.001
BMI			
18.5 ≤ BMI < 23	1.00	Reference	
BMI < 18.5	1.17	(0.820, 1.668)	0.388
23 ≤ BMI < 25	0.98	(0.848, 1.131)	0.774
BMI ≥ 25	1.19	(1.048, 1.355)	0.008
Waist circumference (cm)	1.03	(1.020, 1.032)	<0.001
Income			
Q1	1.00	Reference	
Q2	0.76	(0.655, 0.886)	<0.001
Q3	0.61	(0.521, 0.709)	<0.001
Q4	0.52	(0.442, 0.606)	<0.001
Education			
Less than elementary school	1.00	Reference	
Middle school	0.47	(0.401, 0.558)	<0.001
High school	0.28	(0.234, 0.323)	<0.001
College or higher	0.18	(0.140, 0.241)	<0.001
Alcohol consumption			
Not drinking at all	1.00	Reference	
Less than once a month	0.86	(0.699, 1.059)	0.157
About once a month	0.62	(0.464, 0.838)	0.002
About once a week	0.50	(0.342, 0.727)	<0.001
Almost every day	0.91	(0.465, 1.770)	0.775
Smoking			
Nonsmoker	1.00	Reference	
Smoker	1.15	(0.935, 1.406)	0.188
Oral contraceptive use			
No	1.00	Reference	
Yes	1.36	(1.188, 1.546)	<0.001
Number of days of walking exercise per week			
0 day/week	1.00	Reference	
1–3 days/week	0.51	(0.439, 0.596)	<0.001
≥4 days/week	0.62	(0.539. 0.705)	<0.001
Number of days of strength exercise per week			
0 day/week	1.00	Reference	
1–3 days/week	0.48	(0.388, 0.590)	<0.001
≥4 days/week	0.72	(0.559, 0.928)	0.011
Dietary circumferences			
Eating enough and varied food	1.00	Reference	
Eating enough but not diverse food	1.60	(1.412, 1.803)	<0.001
Eating poorly sometimes	3.33	(2.522, 4.383)	<0.001
Eating poorly frequently	4.48	(2.402, 8.363)	<0.001
Hypertension status			
Normal	1.00	Reference	
Hypertension	1.86	(1.664, 2.080)	<0.001
Diabetes status			
Normal	1.00	Reference	
Diabetes	1.50	(1.276, 1.758)	<0.001
Dyslipidemia status			
Normal	1.00	Reference	
Dyslipidemia	1.41	(1.232, 1.608)	<0.001

Note: OR, odds ratio; CI, confidence interval; Q, quarter; BMI, body mass index.

**Table 3:** Multivariate logistic regression analysis of chronic low back pain risk by chronic disease status categorized into models 1, 2, and 3.

Risk for Chronic Low Back Pain Compared to That of Non-Chronic Low Back Pain									
	Model 1			Model 2			Model 3		
	OR	95% CI	*p*-Value	OR	95% CI	*p*-Value	OR	95% CI	*p*-Value
Hypertension status									
Normal	1.00	Reference		1.00	Reference		1.00	Reference	
Hypertension	1.11	(0.976, 1.259)	0.112	1.11	(0.977, 1.264)	0.107	1.11	(0.970, 1.265)	0.132
Diabetes status									
Normal	1.00	Reference		1.00	Reference		1.00	Reference	
Diabetes	0.99	(0.835, 1.177)	0.921	0.97	(0.818, 1.157)	0.756	0.96	(0.802, 1.148)	0.653
Dyslipidemia status									
Normal	1.00	Reference		1.00	Reference		1.00	Reference	
Dyslipidemia	1.25	(1.088, 1.439)	0.002	1.32	(1.144, 1.522)	<0.001	1.32	(1.140, 1.530)	<0.001

Model 1: adjust for age, weight, height, waist circumference, and oral contraceptive use. Model 2: Model 1 + adjustment for income and education. Model 3: Model 2 + adjustment for number of days of walking exercise per week, number of days of strength exercise per week, and dietary circumferences. Note: OR, odds ratio; CI, confidence interval.

**Table 4:** Multivariate logistic regression analysis of chronic low back pain risk by chronic disease status categorized by BMI into underweight, normal, overweight, and obesity.

Risk for Chronic Low Back Pain Compared to That of Non-Chronic Low Back Pain												
	Underweight (BMI < 18.5)			Normal (18.5 ≤ BMI < 23)			Overweight (23 ≤ BMI < 25)			Obesity (BMI ≥ 25)		
	OR	95% CI	*p*-Value	OR	95% CI	*p*-Value	OR	95% CI	*p*-Value	OR	95% CI	*p*-Value
Hypertension status												
Normal	1.00	Reference		1.00	Reference		1.00	Reference		1.00	Reference	
Hypertension	2.03	(0.732, 5.641)	0.174	0.95	(0.750, 1.205)	0.676	1.10	(0.840, 1.430)	0.500	1.20	(0.970, 1.482)	0.093
Diabetes status												
Normal	1.00	Reference		1.00	Reference		1.00	Reference		1.00	Reference	
Diabetes	1.01	(0.296, 3.463)	0.984	0.71	(0.500, 1.006)	0.054	1.10	(0.744, 1.615)	0.642	1.06	(0.820, 1.380)	0.639
Dyslipidemia status												
Normal	1.00	Reference		1.00	Reference		1.00	Reference		1.00	Reference	
Dyslipidemia	0.20	(0.020, 2.064)	0.178	1.21	(0.908, 1.602)	0.196	1.12	(0.833, 1.501)	0.457	1.64	(1.310, 2.040)	<0.001

Results of multivariate logistic regression analysis adjusted with age, weight, height, waist circumference, OC use, individual income, education level, number of days of walking exercise per week, number of days of strength exercise per week, and dietary circumferences. Note: OR, odds ratio; CI, confidence interval.

**Table 5:** Multivariate logistic regression analysis of chronic low back pain risk by chronic disease status categorized by 60 years.

Risk for Chronic Low Back Pain Compared to That of Non-Chronic Low Back Pain						
	Age < 60 Years			Age ≥ 60 Years		
	OR	95% CI	*p*-Value	OR	95% CI	*p*-Value
Hypertension status						
Normal	1.00	Reference		1.00	Reference	
Hypertension	1.11	(0.855, 1.430)	0.444	1.11	(0.946, 1.296)	0.206
Diabetes status						
Normal	1.00	Reference		1.00	Reference	
Diabetes	0.81	(0.510, 1.271)	0.351	0.99	(0.819, 1.219)	0.993
Dyslipidemia status						
Normal	1.00	Reference		1.00	Reference	
Dyslipidemia	1.61	(1.235, 2.090)	<0.001	1.31	(1.090, 1.565)	0.004

Results of multivariate logistic regression analysis adjusted with age, weight, height, waist circumference, OC use, individual income, education level, number of days of walking exercise per week, number of days of strength exercise per week, and dietary circumferences. Note: OR, odds ratio; CI, confidence interval.

## Data Availability

Data and materials are available on reasonable request. The raw data of KNHANES used in this paper can be accessed through the following website. https://knhanes.kdca.go.kr/knhanes/sub03/sub03_02_05.do (accessed on 1 September 2023).
